# Supplementary figures and images for: A Modular, Cost-Effective, and Pumpless Perfusion Assembly for the Long-Term Culture of Engineered Microvessels
Source: Micromachines (Basel). 2025 Mar 19;16(3):351. doi: 10.3390/mi16030351 (PMC11945127; doi:10.3390/mi16030351)

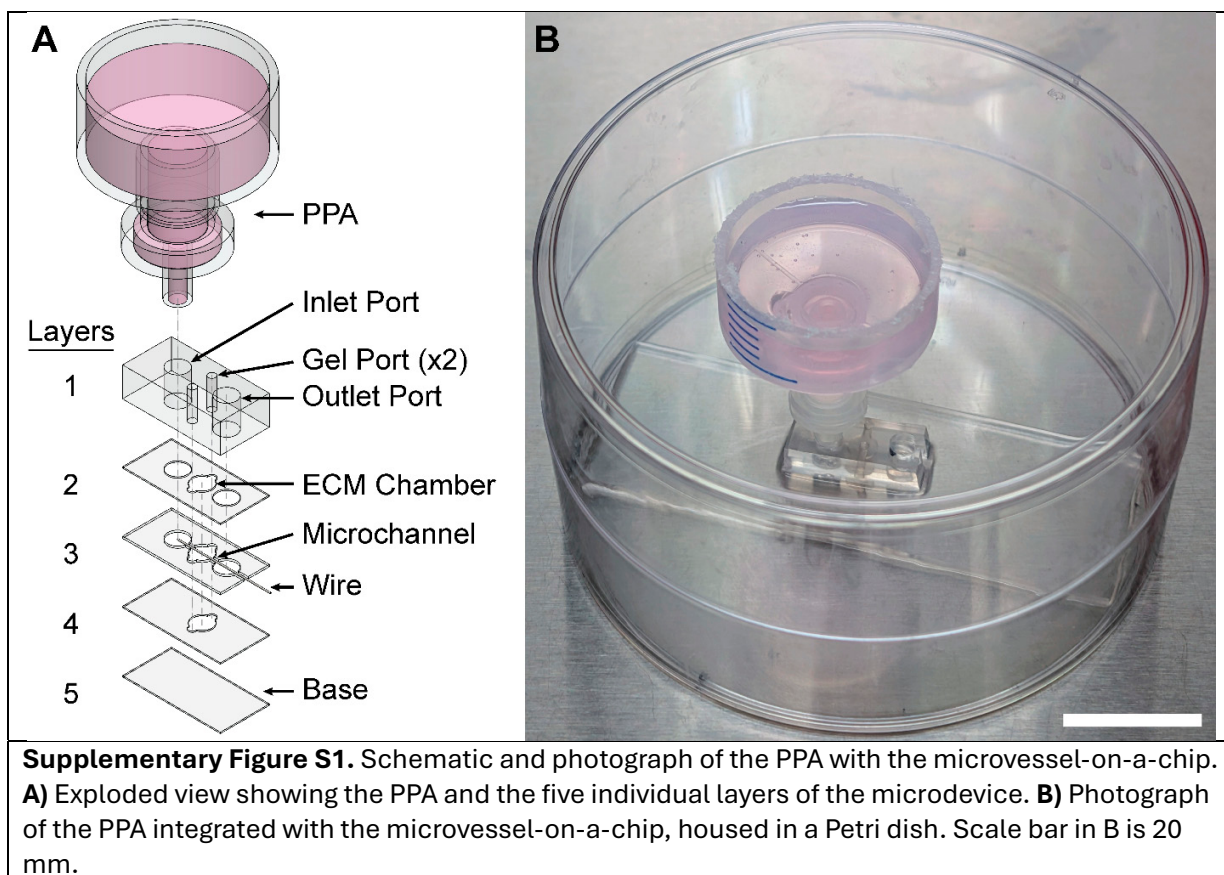

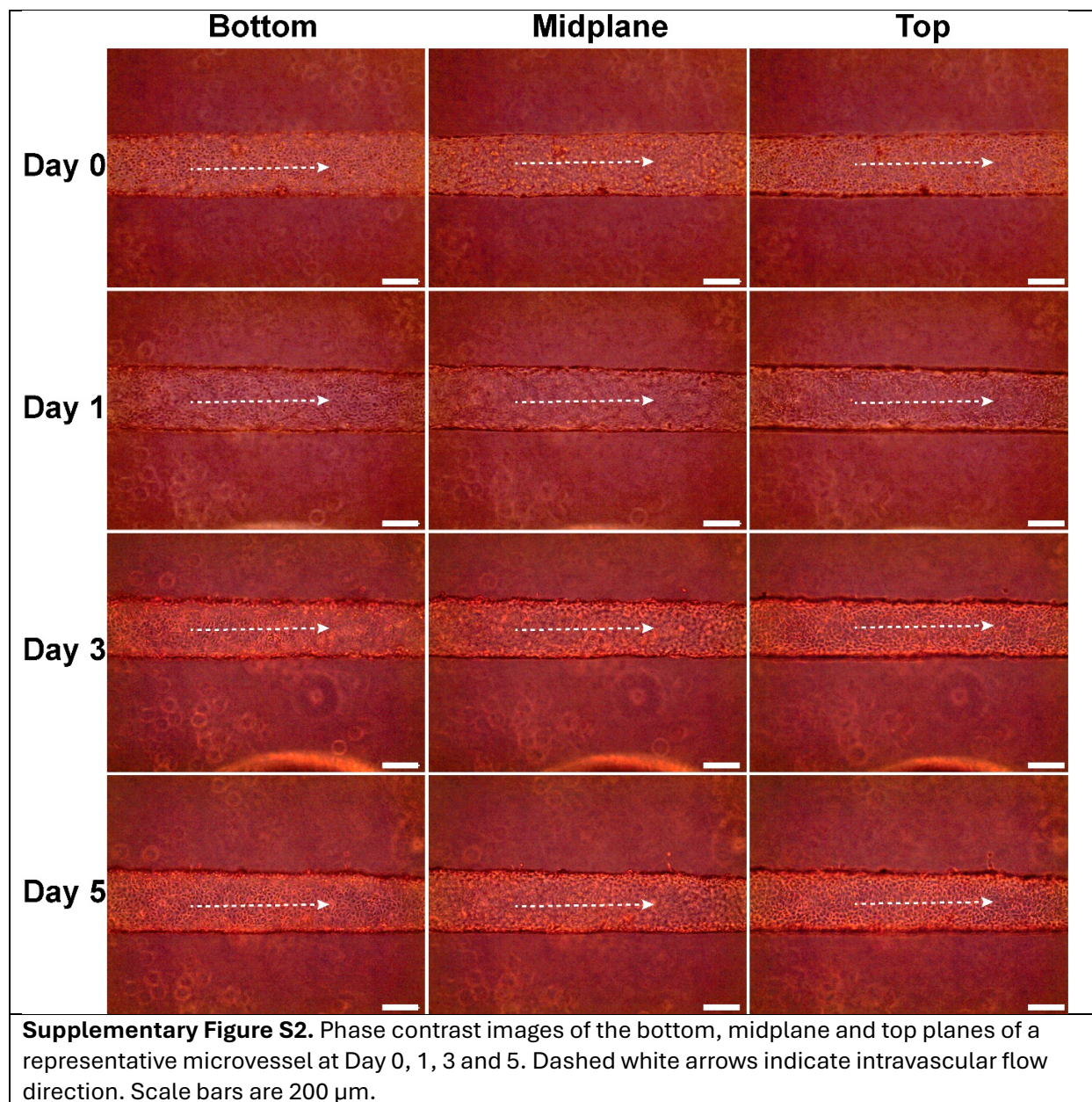

Supplement: Supplementary file 1 [file micromachines-16-00351-s001.zip › micromachines-3490370-supplementary.pdf]
